# Synthesis of Cobalt-Substituted Manganese Phosphate Purple Pigments

**DOI:** 10.3390/ma16114132

**Published:** 2023-06-01

**Authors:** Saki Aso, Hiroaki Onoda

**Affiliations:** Department of Informatics and Environmental Sciences, Kyoto Prefectural University, 1-5 Shimogamo Nakaragi-cyo, Sakyo-ku, Kyoto 606-8522, Japan; s822631001@kpu.ac.jp

**Keywords:** inorganic pigment, phosphate material, acid and base resistances

## Abstract

Some manganese phosphates are known as violet pigments. In this study, pigments in which manganese was partially replaced with cobalt and aluminum was replaced with lanthanum and cerium were synthesized with a heating method to obtain pigments with a more reddish color. The obtained samples were evaluated in terms of chemical composition, hue, acid and base resistances, and hiding power. Among the samples examined, the samples obtained in the Co/Mn/La/P system were the most vivid. The brighter and redder samples were obtained by prolonged heating. Furthermore, prolonged heating improved the acid and base resistance of the samples. Finally, the substitution of manganese for cobalt improved the hiding power.

## 1. Introduction

Inorganic pigments have better resistance to heat and weather than organic pigments [1]. Therefore, they are widely used in applications where durability is strongly required, such as coloring ceramics and plastics, and pain for cars, buildings, and houses [2,3]. However, one of the characteristics of inorganic pigments is that they have low coloring power and cause rapid sedimentation in nonpolar media because of their high density [4]. In particular, inorganic purple pigments easily absorb ultraviolet rays, and chemical changes such as oxidation cause them to become a different substance, which tends to fade [5]. In order to solve this problem, inorganic pigments must be chemically stable, and therefore often contain toxic metals such as cadmium compounds [6]. In fact, pigments made with cadmium compounds have high color retention and are bright and suitable for warm colors; however, they are extremely toxic and have negative effects on the human body and the environment. The World Health Organization has pointed out that paints containing pigments prepared with harmful metals such as cadmium compounds are used to paint buildings and toys, and 143,000 people die and more than 600,000 children are mentally disabled due to inhalation of deteriorated powders every year [7]. Therefore, it is necessary to solve these problems through the development of novel inorganic pigments that are friendly to the human body and the environment without containing harmful metals. As inorganic pigments, manganese compounds are possible candidates for purple pigments [8]. In general, manganese compounds with tetravalent manganese tend to be black (for example MnO_2_), trivalent compounds are purple, and divalent compounds are light pink. In other words, trivalent manganese compounds can be expected to produce a purple pigment that fits the purpose.

A previous study has used manganese compounds with relatively low environmental impact to produce vivid inorganic purple pigments that do not contain harmful metals [9]. More specifically, it has been reported that a manganese compound, aluminum compound, and phosphoric acid enables the synthesis of slightly more deeply vivid violet pigments [10].

In inorganic compounds, cobalt compounds are suitable for enhancing cold colors such as blueness, and it is known that they may cause granulation while having slightly fewer toxic characteristics than cadmium compounds [11,12,13,14]. Cobalt ions are present in red colors when divalent because hexagonal cobalt (II) ions coordinated to water are red [15]. Since these ions have a tendency to turn blue when anhydrous, the oxidation of cobalt ions must be controlled to achieve a well-balanced composition to produce a vivid purple pigment [16]. In addition, rare earth compounds generally have low solubility and are thought to contribute to the production of pigments that are resistant to acids and bases [17]. Among the rare earth elements, lanthanum and cerium are relatively abundant and readily available, and their chemical properties differ due to their different valences. Therefore, these two rare earth elements were examined.

Based on the above, in this study we attempted to synthesize a novel purple pigment that was safe for the human body and the environment by using a heating method [10]. Since the heating method enables the formation of target compounds and their crystal growth, it was considered that more vivid purple pigments could be generated. In addition to further expanding knowledge on the production of purple pigments obtained in previous research, the use of the cobalt compound was expected to produce novel inorganic purple pigments with further enhanced functionality.

## 2. Materials and Methods

Manganese oxide, MnO_2_, was mixed with aluminum hydroxide, Al(OH)_3_, in the molar ratio Mn/Al = 1/1. This mixture was added to 10 mL of water and 2 mL of phosphoric acid (14 mol/L) so that Mn/Al/P = 1/1/4. The mixture was allowed to stand for one day in the mullite crucible, and then heated at 400 °C for 1 h by an electric furnace. The heating times and temperatures were determined from previous studies [9,10]. In this sample preparation, some of the manganese oxide was replaced by cobalt oxide, Co_3_O_4_ (Co/Mn = 0.2/0.8, 0.5/0.5, 0.8/0.2, 1/0), and heated in the same manner. In addition, samples in which aluminum hydroxide was replaced by lanthanum oxide, La_2_O_3_, or cerium carbonate, Ce_2_(CO_3_)_3_•8H_2_O, were also prepared. Samples with lanthanum oxide were also prepared by heating for 20 h. In previous studies, an extension of the heating time to 20 h resulted in an increase in the vividness of the samples [18]. Therefore, for conditions that showed relatively good results, samples were prepared even at 20 h.

The crystalline phase composition of these materials was analyzed with X-ray diffraction (XRD; MiniFlex; Rigaku Corp., Akishima, Japan) using a monochromatic Cu-Kα ray. Infrared (IR) spectra were recorded with the KBr disk method using a HORIBA FT-IR 720 (Horiba Ltd., Kyoto, Japan).

The color of phosphate pigments was estimated from ultraviolet–visible (UV–Vis) reflectance spectra acquired using a Shimadzu UV2100 (Kyoto, Japan) (reference compound: BaSO_4_). The color of the samples was evaluated using a TES135 plus color analyzer (TES Electrical Electronic Corp, Taipei, Taiwan). The L* value refers to the lightness of the powder, where 100 is white and its opposite, 0, is black. The a* value refers to the redness of the material, where positive values correspond to red and negative values to green. The b* value refers to the yellowish color, where positive values correspond to yellow and negative values to blue, respectively. While L*a*b* values are good for simple comparisons of color differences, UV–visible reflectance spectra provide a fine-grained change in each wavelength.

For the evaluation of acid and base resistance, samples (0.1 g) were exposed to 0.1 wt% sulfuric acid (100 mL) or 0.1 wt% sodium hydroxide solution (100 mL) for 24 h. Sulfuric acid was used as a common, non-volatile acid. The concentrations and durations were determined to avoid dissolving all or none of the sample.

Finally, the hiding power was evaluated. In paints generally available on the market, linseed oil is used as a medium for dark colors such as red, brown, and black, and poppy oil for white and light colors [19,20]. Since the color of the pigment prepared in this work was a deep purple, 1.38 g of sun-thickened linseed oil and 0.167 g of stand linseed oil were used as drying oils, and 0.05 g of pure beeswax and 0.05 g of dammar gum were also used as resins. The 0.016 g of *siccative courtrai* as an agent for drying and 0.5 g of the sample were finally mixed while heating from 50 °C to 60 °C to prepare a paint. The resulting paint was then applied to the lettered canvas, confirming its hiding power.

## 3. Results and Discussion

**Figure 1:**
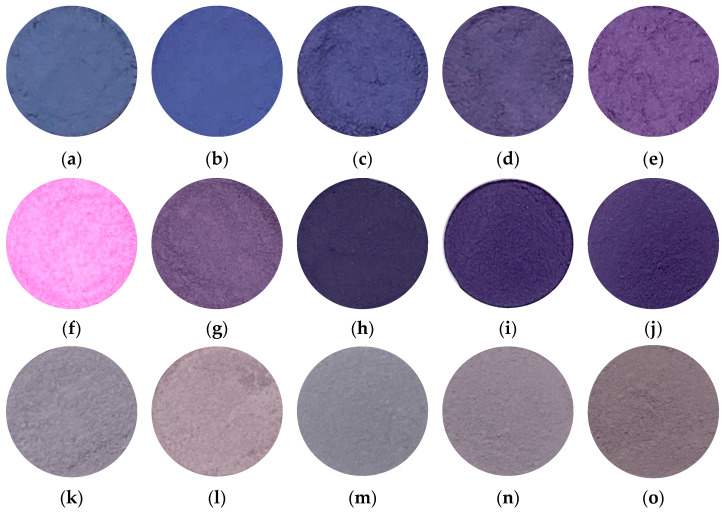
Photographs of samples prepared in various conditions (400 °C, 1 h), Co/Mn/Al/P = x/(1 − x)/1/4, (**a**) x = 1, (**b**) 0.8, (**c**) 0.5, (**d**) 0.2, (**e**) 0, Co/Mn/La/P = x/(1 − x)/1/4, (**f**) x = 1, (**g**) 0.8, (**h**) 0.5, (**i**) 0.2, (**j**) 0, Co/Mn/Ce/P = x/(1 − x)/1/4, (**k**) x = 1, (**l**) 0.8, (**m**) 0.5, (**n**) 0.2, and (**o**) 0.

Figure 1 shows the photographs of samples prepared in various conditions. Samples prepared in Co/Mn/Al/P = 1/0/1/4 and 0.8/0.2/1/4 (Figure 1a,b) exhibited a deep blue color. Therefore, in samples prepared with the aluminum compound (Figure 1a–e), the lower the percentage of cobalt, the stronger the redness. Since divalent manganese ions tend to be pale pink, and trivalent manganese ions tend to be purple compounds, the purple color gradually became stronger as the proportion of trivalent manganese increased. Considering this, in these samples it could be inferred that manganese in the sample existed as trivalent manganate ions. All of the samples prepared with the lanthanum compound (Figure 1f–j) exhibited vivid colors, and therefore it was expected that the composition of cobalt, lanthanum, and phosphoric acid would enable the production of a vivid purple pigment. In particular, the sample prepared in Co/Mn/La/P = 1/0/1/4 (Figure 1f) exhibited a vivid purple color. In addition, even if a part of the cobalt cations was replaced with safer manganese cations, sample powders were able to keep the purple/violet color. Therefore, it was considered that a safer purple/violet pigment could be produced via manganese substitution. The violet hue was maintained by replacing manganese ions because trivalent manganese phosphate tends to give a violet color. Most of samples prepared with the cerium compound (Figure 1k–o) were refined with a strong gray tint, and it was found that cerium compounds were not suitable for the production of purple pigments.

**Figure 2:**
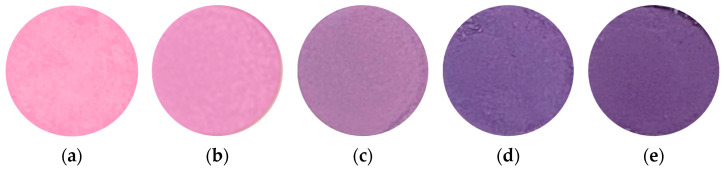
Photographs of samples prepared in various conditions (400 °C, 20 h), Co/Mn/La/P = x/(1 − x)/1/4, (**a**) x = 1, (**b**) 0.8, (**c**) 0.5, (**d**) 0.2, and (**e**) 0.

Figure 2 shows the photographs of samples prepared in various conditions. It can be seen that by heating for 20 h, brighter purple-violet pigments were produced than in samples produced by heating for 1 h. For samples heated for 1 h, as described above, it was observed that the more the cobalt ratio decreased, the darker the color of the samples became. The same tendency was observed for the sample heated for 20 h. The reason for this change was considered to be the coordination number of water to cobalt being reduced. Since the hue of cobalt changes depending on the coordination number, it can be thought of as having a purple hue when cobalt is 6-coordinated in the pigment [15]. These results indicated that the presence of cobalt is an important factor in the formation of bright purple-violet pigments.

Table 1 shows the L*a*b* values of the samples prepared in various conditions. The L*a*b* values could be said to show a bright purple pigment, as the a* value increased in the positive direction and the b* value increased in the negative direction. Overall, the samples had strong reddish and bluish tinges, and had a suitable balance between a* and b* values; therefore, it can be said that it was suitable as a purple pigment. Most of the samples heated for 20 h showed improved redness and bluishness compared to those heated for 1 h. From this, it became clear that the extension of the heating time was one of the factors for improving the color of the sample. There was no relationship between the ΔE*ab values calculated from the samples heated for 1 and 20 h and the x values.

**Figure 3:**
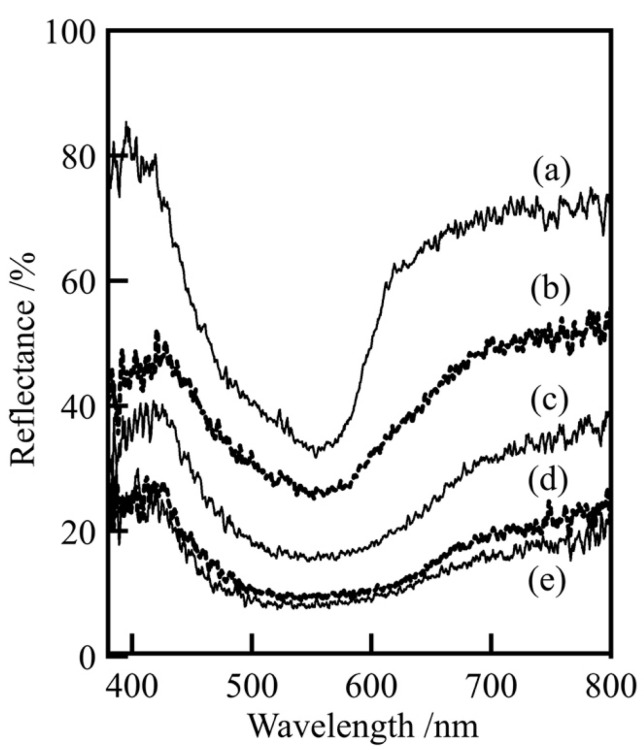
UV–Vis. reflectance spectra of samples prepared in various conditions, Co/Mn/La/P = x/(1 − x)/1/4, (**a**) x = 1, (**b**) 0.8, (**c**) 0.5, (**d**) 0.2, and (**e**) 0 (400 °C, 20 h).

Figure 3 shows the UV–visible reflectance spectra of samples prepared in various conditions. It can be seen that the samples exhibited a purple color because of the peaks at 380–450 nm. In addition, since samples prepared in Co/Mn/La/P = 1/0/1/4, 0.8/0.2/1/4 and 0.5/0.5/1/4 had the strong absorption peaks at this area (Figure 3a–c), it was found that the samples with a high cobalt ratio could be expected to have improved vividness. On the other hand, when the value of x varied from 0.5 to 0, no significant change was observed. This indicates that the presence ratio of cobalt had a significant effect on the hue, with higher presence ratios resulting in more vivid colors.

**Figure 4:**
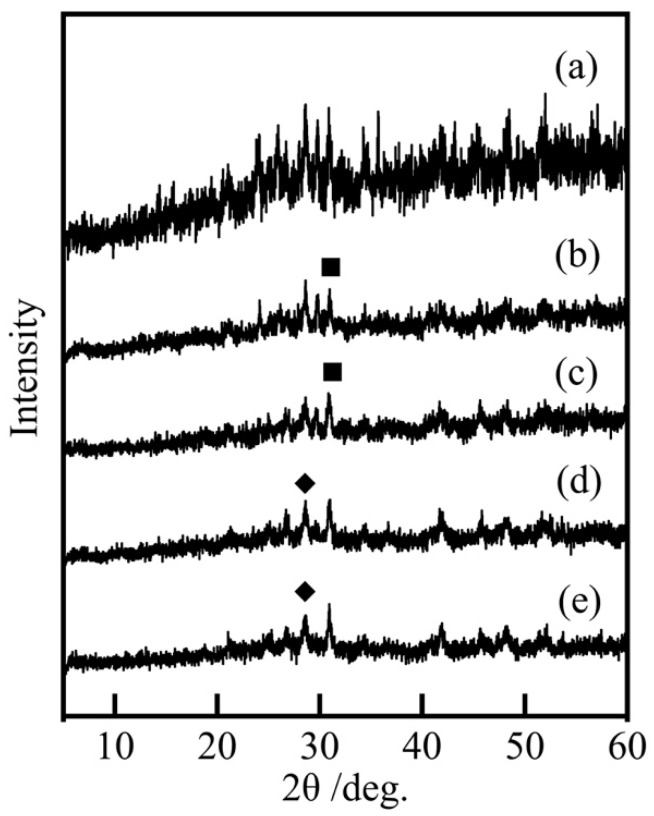
XRD patterns of samples prepared in various conditions (400 °C, 20 h), Co/Mn/La/P = x/(1 − x)/1/4, (**a**) x = 1, (**b**) 0.8, (**c**) 0.5, (**d**) 0.2, and (**e**) 0, ◆: Mn_2_P_2_O_7_, ■: H_4_Co_7_(PO_4_)_6_.

Figure 4 shows the XRD patterns of samples prepared in various conditions. The 28.5° and 31° peaks were attributed to Mn_2_P_2_O_7_ and H_4_Co_7_(PO_4_)_6_, respectively. In addition to these peaks, multiple peaks were observed and many raw material compositions were used; therefore, it could be considered that compounds other than Mn_2_P_2_O_7_ and H_4_Co_7_(PO_4_)_6_ were also formed. The presence of these multiple compounds resulted in overall lower peaks, making it difficult to identify the compounds. This was thought to be due to the relatively low temperature and the use of multiple cations, which made it difficult for crystals to form. As a result, these compounds had low crystallinity and were close to amorphous. However, purple-violet pigments were obtained under all conditions, and it can be assumed that the coexistence of many compounds resulted in the appropriate hue.

**Figure 5:**
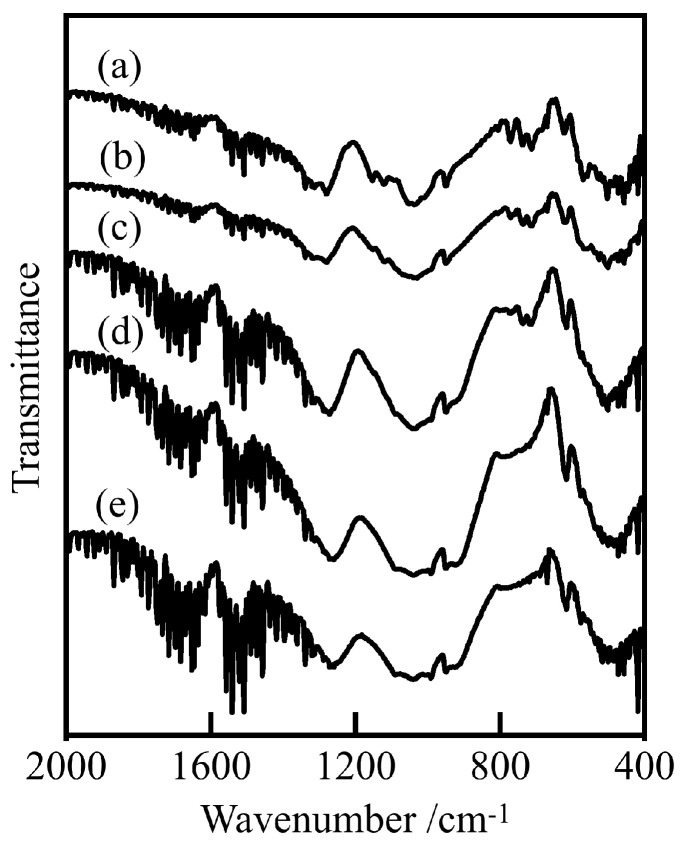
FT-IR spectra of samples prepared in various conditions (400 °C, 20 h), Co/Mn/La/P = x/(1 − x)/1/4, (**a**) x = 1, (**b**) 0.8, (**c**) 0.5, (**d**) 0.2, and (**e**) 0.

Figure 5 shows the FT-IR spectra of samples prepared in various conditions. All samples showed peaks at 500, 900–1100, and 1280 cm^−1^. There was a bending vibration of the O-P-O bond, which was confirmed from 500 cm^−1^, and the stretching vibration of the P-O bond and the P=O bond, which were the peaks near 900–1100 cm^−1^ and 1280 cm^−1^, respectively. From this, it was confirmed that all the samples contained phosphate [21]. The higher the cobalt ratio, the larger the P-O bond peak around 900–1100 cm^−1^.

**Figure 6:**
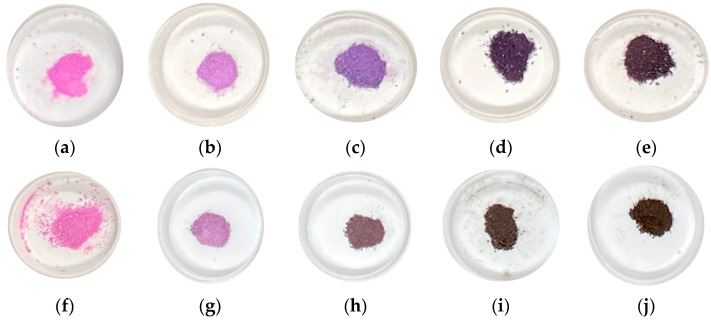
Photographs of samples heated at various ratios and then exposed to 0.1 wt% sulfuric acid solution and 0.1 wt% sodium hydroxide solution for 24 h, (acid-exposed) Co/Mn/La/P = x/(1 − x)/1/4, (**a**) x = 1, (**b**) 0.8, (**c**) 0.5, (**d**) 0.2, (**e**) 0, (base-exposed) (**f**) x = 1, (**g**) 0.8, (**h**) 0.5, (**i**) 0.2, and (**j**) 0.

Figure 6 shows photographs of samples when acid and base resistances were evaluated. The original samples exposed to acidic and basic solutions are indicated by acid-exposed samples (Figure 6a–e) and base-exposed samples (Figure 6f–j), respectively. In samples exposed to the acidic solution, no significant decolorization was observed. In particular, samples prepared in Co/Mn/La/P = 1/0/1/4, 0.8/0.2/1/4 and 0.5/0.5/1/4 kept their purple color and retained the vividness of the original sample (Figure 6a–c). The similar results described above were obtained when exposed to a basic solution. Compared with the samples heated for 1 h when exposed to a basic solution, it was found that the base resistance was enhanced by extending the heating hours. In addition, the overall results showed that the acid and base resistances were improved as the proportion of cobalt increased.

Table 2 and Table 3 show the L*a*b* and ΔE*ab values and the yields of various samples after exposures to acidic and basic solutions. Since samples of Co/Mn/La/P = 1/0/1/4 and 0.8/0.2/1/4 had positive a* values and negative b* values, the purple color of the samples was retained even when exposed to acidic or basic solutions. The larger the ΔE*ab values, the more discolored the original sample, which meant it was considered to be unsuitable for practical use. Samples with a high ratio of cobalt indicated smaller ΔE*ab values. This indicates that the cobalt substitution is an important factor in maintaining the original color. The yields of samples after exposure to acidic or basic solutions for 24 h was 67% or more, which could be evaluated as a tolerance of the sample for acidic or basic solutions.

**Figure 7:**
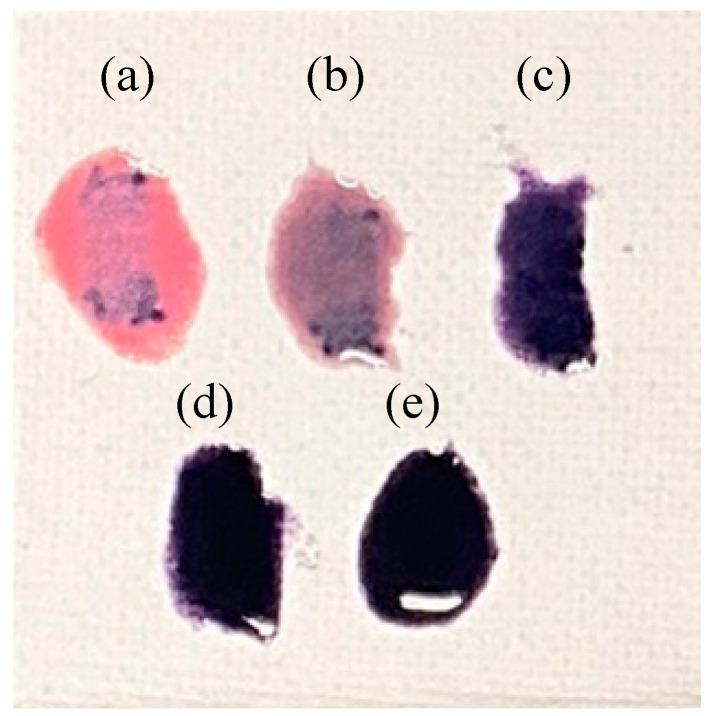
Photographs of paints on a cotton canvas, Co/Mn/La/P = x/(1 − x)/1/4, (**a**) x = 1, (**b**) 0.8, (**c**) 0.5, (**d**) 0.2, and (**e**) 0 (400 °C, 20 h).

Figure 7 shows the photographs showing the hiding power of the prepared samples. In particular, in the sample of Co/Mn/La/P = 1/0/1/4, oil paint was produced in a state in which the powder did not blend well with the oil and separated. Comparing the prepared paints, the samples of Co/Mn/La/P = 0.5/0.5/1/4, 0.2/0.8/1/4, and 0/1/1/4 (Figure 7c–e) were relatively stable with the oil, and the color of the mixed sample with the oil did not deteriorate. Conversely, the stronger the redness, the significantly poorer the coloring and hiding power. The reason that the *sun-thickened linseed oil* was a yellow oil was because if the color of the sample was not deep, the color of the paint loses to yellowness. Light color powders such as the samples prepared in Co/Mn/La/P = 1/0/1/4 and 0.8/0.2/1/4 (Figure 7a,b) may need to be manufactured using a coloring agent, for example poppy oil or safflower oil.

## 4. Conclusions

The novel phosphate pigments were prepared by heating at various Co/Mn/(Al, La, Ce)/P ratios. By controlling the composition and heating time, bright purple pigments could be obtained. All samples were low crystalline phosphates. By increasing the heating time and substituting with cobalt, pigments resistant to acids and bases were obtained. It was found that as the ratio of cobalt increased, the color of the powder became lighter and the hiding power decreased. In this study, inorganic phosphate pigments with brilliant colors were obtained using relatively low toxicity compounds.

## Figures and Tables

**Table 1:** L*a*b* values of samples prepared in various Co/Mn/La/P = x/(1 − x)/1/4 ratios (400 °C).

		1 h			20 h		ΔE*ab
x	L*	a*	b*	L*	a*	b*	
1	75.1	12.6	−9.2	66.5	20.0	−12.5	11.8
0.8	56.1	15.3	−6.3	63.4	11.2	−7.4	8.4
0.5	31.9	5.4	−2.9	47.6	12.4	−15.2	21.1
0.2	34.9	6.7	−5.5	35.4	10.5	−15.3	10.5
0	49.6	−9.0	−10.7	36.2	12.1	−13.7	25.2

1 h

20 h

ΔE*ab

**Table 2:** L*a*b* values and yields of samples prepared in various Co/Mn/La/P = x/(1 − x)/1/4 ratios (400 °C, 20 h) and then exposed to 0.1 wt% sulfuric acid solution for 24 h.

x	L*	a*	b*	ΔE*ab	Yield/%
1	69.1	24.4	−8.0	6.8	84
0.8	61.8	9.7	−4.7	3.5	80
0.5	67.9	7.0	−3.2	24.2	75
0.2	65.8	−3.9	5.3	39.4	75
0	62.8	1.2	6.8	35.3	78

**Table 3:** L*a*b* values and yields of samples prepared in various Co/Mn/La/P = x/(1 − x)/1/4 ratios (400 °C, 20 h) and then exposed to 0.1 wt% sodium hydroxide solution for 24 h.

x	L*	a*	b*	ΔE*ab	Yield/%
1	72.2	22.5	−5.6	9.3	87
0.8	53.2	15.3	−7.0	11.0	79
0.5	55.9	−0.9	6.4	26.7	77
0.2	77.2	7.0	−3.9	43.5	67
0	66.4	−4.3	10.4	42.0	76

## Data Availability

Not applicable.
